# Multi-arm multi-stage (MAMS) randomised selection designs: impact of treatment selection rules on the operating characteristics

**DOI:** 10.1186/s12874-024-02247-w

**Published:** 2024-06-03

**Authors:** Babak Choodari-Oskooei, Alexandra Blenkinsop, Kelly Handley, Thomas Pinkney, Mahesh K. B. Parmar

**Affiliations:** 1 MRC Clinical Trials Unit at UCL, Institute of Clinical Trials and Methodology, UCL, 90 High Holborn, WC1V 6LJ London, United Kingdom; 2 Department of Mathematics, Imperial College London, London, UK; 3 Birmingham Clinical Trials Unit, University of Birmingham, Birmingham, UK; 4 Institute of Applied Health Research, University of Birmingham, Birmingham, UK

**Keywords:** Multi-arm multi-stage randomised clinical trials, MAMS, Treatment selection, Adaptive trial designs, Familywise type I error rate, ROSSINI-2 trial

## Abstract

**Background:**

Multi-arm multi-stage (MAMS) randomised trial designs have been proposed to evaluate multiple research questions in the confirmatory setting. In designs with several interventions, such as the 8-arm 3-stage ROSSINI-2 trial for preventing surgical wound infection, there are likely to be strict limits on the number of individuals that can be recruited or the funds available to support the protocol. These limitations may mean that not all research treatments can continue to accrue the required sample size for the definitive analysis of the primary outcome measure at the final stage. In these cases, an additional treatment selection rule can be applied at the early stages of the trial to restrict the maximum number of research arms that can progress to the subsequent stage(s).

This article provides guidelines on how to implement treatment selection within the MAMS framework. It explores the impact of treatment selection rules, interim lack-of-benefit stopping boundaries and the timing of treatment selection on the operating characteristics of the MAMS selection design.

**Methods:**

We outline the steps to design a MAMS selection trial. Extensive simulation studies are used to explore the maximum/expected sample sizes, familywise type I error rate (FWER), and overall power of the design under both binding and non-binding interim stopping boundaries for lack-of-benefit.

**Results:**

Pre-specification of a treatment selection rule reduces the maximum sample size by approximately 25% in our simulations. The familywise type I error rate of a MAMS selection design is smaller than that of the standard MAMS design with similar design specifications without the additional treatment selection rule. In designs with strict selection rules - for example, when only one research arm is selected from 7 arms - the final stage significance levels can be relaxed for the primary analyses to ensure that the overall type I error for the trial is not underspent. When conducting treatment selection from several treatment arms, it is important to select a large enough subset of research arms (that is, more than one research arm) at early stages to maintain the overall power at the pre-specified level.

**Conclusions:**

Multi-arm multi-stage selection designs gain efficiency over the standard MAMS design by reducing the overall sample size. Diligent pre-specification of the treatment selection rule, final stage significance level and interim stopping boundaries for lack-of-benefit are key to controlling the operating characteristics of a MAMS selection design. We provide guidance on these design features to ensure control of the operating characteristics.

**Supplementary Information:**

The online version contains supplementary material available at 10.1186/s12874-024-02247-w.

## Introduction

Multi-arm multi-stage (MAMS) trial designs can efficiently evaluate several medical interventions by allowing multiple research arms to be studied under one protocol and enabling interim stopping for lack-of-benefit based on primary (or an intermediate) outcome measure of the trial. In MAMS designs, the research arms are compared against a common control arm (generally, standard-of-care treatment) and these pairwise comparisons can be made in several stages. Royston et al. developed a framework for a MAMS design that allows the use of an intermediate (*I*) outcome at the interim stages that may or may not be the same as the definitive (*D*) outcome at the final analysis [1–3]. Choodari-Oskooei et al. give an extensive account of Royston et al.’s MAMS design and discuss their underlying principles [3].

In the Royston et al. standard MAMS design, monotonically decreasing significance levels are defined for the interim-stage lack-of-benefit analyses to determine which research interventions can continue recruiting patients [2]. In principle, all research arms which perform sufficiently better than the control arm at each interim analysis, by a pre-defined threshold, can continue recruitment and have the potential to reach the final stage efficacy analysis. This approach to treatment selection has been described as a *keep all promising* ‘rule’ [4]. However, two challenges may arise under such a framework. First, the maximum sample size, which is achieved when all arms reach the final stage, might become too large if the study includes several research treatment arms. Therefore, the maximum sample size of the standard MAMS design with the *keep all promising* rule can become unfeasible in settings where the resources (e.g patients/funding) are limited. An example is the ROSSINI-2 trial in surgery - see next section for details [5, 6]. Second, there will be large variation in the actual sample size of the trial, depending on how many research arms pass the interim lack-of-benefit analyses. In practice, funders may find it highly desirable to avoid such an uncertainty about the required sample size.

In some settings, there is likely to be a limit on the number of individuals that can be recruited, or the funds available to undertake the protocol. The timeline for a standard (or full) MAMS trial might also be specifically restricted. These constraints can mean not all research treatments can accrue sufficient individuals for the analysis of the primary outcome measure. Therefore, it is highly desirable to consider an additional ‘selection rule’ that determines the maximum number of research arms at each stage, which we henceforth denote a *MAMS selection design*. This would allow the treatment selection and confirmatory stages to be done under the same master protocol, and provide greater control over the overall sample size and required resources. Furthermore, the MAMS selection design formally allows for interim lack-of-benefit stopping and selection rules based on an intermediate outcome measure [7]. This offers higher degrees of flexibility and efficiency compared with alternative designs [8].

This paper addresses several research questions around designing a MAMS trial implementing interim treatment selection rule and allows for interim lack-of-benefit analysis. Previous drop-the-loser designs only allow for interim treatment selection rules [9], whereas the MAMS selection designs of this article allow for both interim teratment selection rule and lack-of-benefit analysis on the primary or intermediate outcome measures [7]. The overarching aim is to show how the maximum (and expected) sample size of a MAMS trial can be reduced by implementing an additional treatment selection rule using a pragmatic approach whilst maintaining desirable overall type I error rate and power. It explores the impact of the number of arms selected (selection rule), the timing of treatment selection together with the chosen threshold for lack-of-benefit analysis on the maximum/expected sample sizes, familywise type I error rate (FWER), and overall power of the design. Finally, it provides practical guidance on how a MAMS selection design can be realised and implemented in trials with several research arms and multiple stages, and to illustrate the advantages of such designs in reducing the required resources.

## Example: ROSSINI-2 selection design

**Fig. 1:**
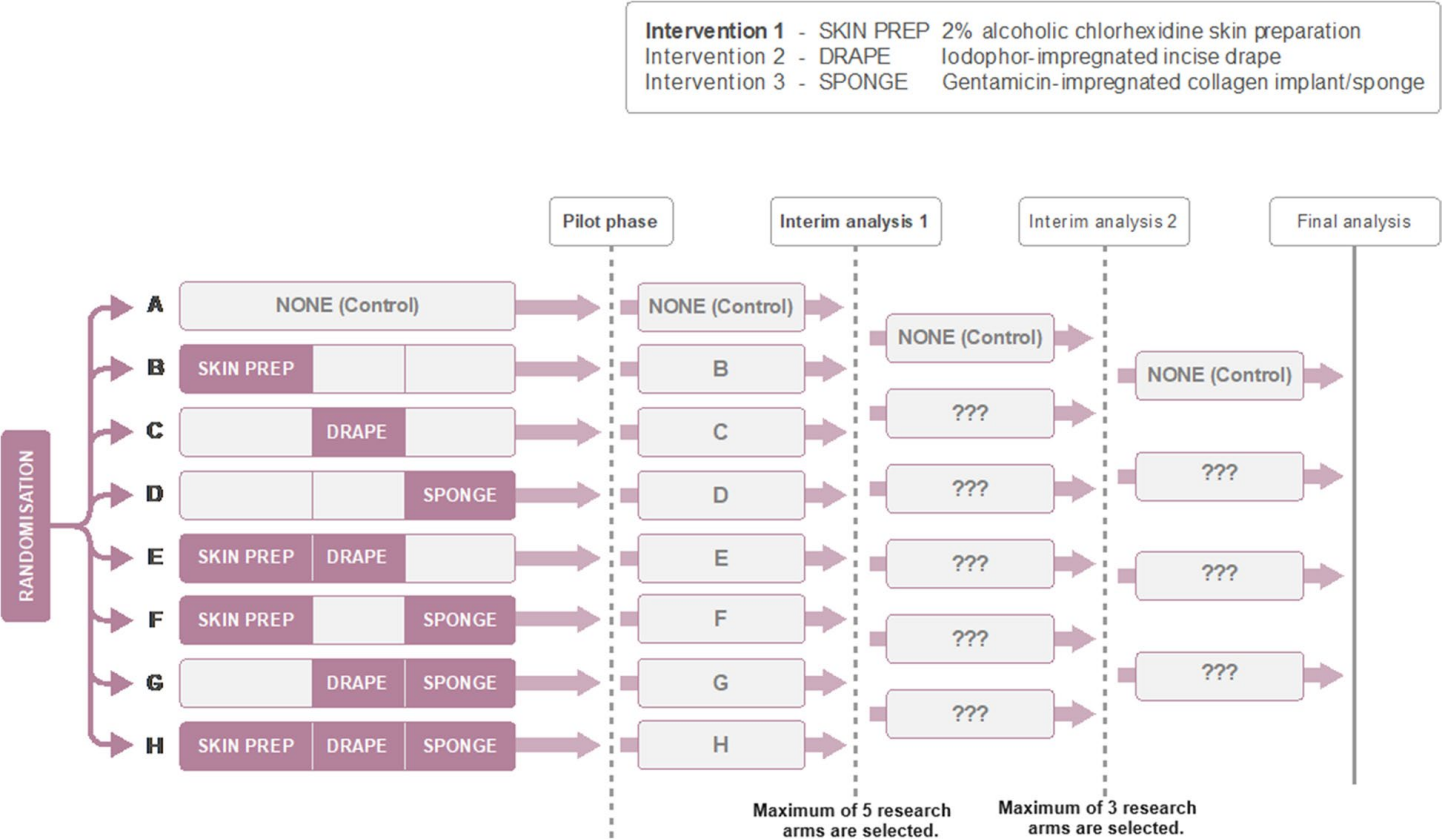
Schema for the ROSSINI-2 MAMS selection design. At least 2 research arms are dropped at each interim stage [6]

Trial setting: The Reduction Of Surgical Site Infection using several Novel Interventions (ROSSINI)-2 trial [NCT03838575] is a phase III 8-arm, 3-stage adaptive design investigating in-theatre interventions to reduce surgical site infection (SSI) following abdominal surgery [5, 6]. In this trial, three interventions are being tested, with patients being randomised to receive all, none or some of these in combination with 7 research arms in total. The control arm is no intervention. A schema of the trial design is represented by Fig. 1 [6]. At the design stage, there was a biological rationale for the single interventions to interact when they are used in combination. But there was no information on the degree of this presumed interaction effect. This ruled out a factorial design for this study.

Design specification: The treatment effect size (used in all stages) is the difference in proportion of patients who develop SSI up to 30 days after surgery. The target effect size is 5% absolute reduction in the SSI event rate in each of the 7 research arms from the control arm event rate of 15%. Patients are randomised with a 2:1 ratio throughout all stages in favour of the control arm - see the online Supplemental Material for more details. The fixed allocation ratio of 2:1 is important since changing the allocation ratio for a particular comparison midcourse a trial implicitly affects the variance of the estimated treatment effect of interest for that comparison, hence potentially violating the equal variance assumption across all comparisons.

Table 1 shows the design parameters for the ROSSINI-2 trial without a selection rule. This is an optimal design which is optimised for a standard MAMS under certain conditions, minimising a loss function - see [10] and online Supplemental Material for details. We used the nstagebinopt and nstagebin Stata commands for this purpose [10]. This standard MAMS design includes two interim lack-of-benefit analyses with interim one-sided significance levels of (0.40, 0.14), acting as the corresponding lack-of-benefit boundaries on the *P*-value scale - i.e., no formal stopping rule for early evidence of efficacy. In the ROSSINI-2 trial, the familywise type I error rate (FWER) is the overall type I error rate of interest since the combination treatments, which included the single interventions, could not be regarded as distinct therapies [11]. The FWER is controlled at 2.5% level (one-sided).

**Table 1:** Design specification for an 8-arm 3-stage design, the ROSSINI-2 trial, without a selection rule. In this standard MAMS design, the overall or maximum sample size assumes all arms reaching the final stage

		Stagewise operating characteristics		Max. sample size^a^
Stage (*j*)	Analysis	Design power ($\omega _{j}$)	Sig. level ($\alpha _{j}$,1-sided)	(Standard MAMS)
1	LOB^b^	0.94	0.40	2358
2	LOB	0.94	0.14	4995
3	efficacy	0.91	0.005	8847
Overall pairwise power^c^		0.85		
Overall FWER^d^ (one-sided)		0.025		

^a^Maximum sample size for the standard MAMS design if research arms pass the previous interim lack-of-benefit analysis. The figures include 4% loss-to-follow-up for the primary outcome - see nstagebin Stata code in Appendix B

^b^LOB, lack-of-benefit

^c^ Pairwise power for the standard MAMS design: Probability of correctly concluding efficacy at final primary analysis for each pairwise comparion against the control arm

^d^ FWER, familywise type I error rate

The maximum sample size of 8847 for this (optimal) standard MAMS design exceeded the budget of the funding agency. Therefore, the trial planned to restrict the number of research arms recruiting at each stage to a maximum of 5 arms in stage 2 and 3 research arms in the final stage - that is, an additional treatment selection rule of 7:5:3, ensuring a maximum sample size of 6613.

## Methods

### Specification of a MAMS selection design

This section outlines the specification of MAMS selection designs, focusing on superiority trials. We assume that the same primary outcome is used at the interim stages for both treatment selection and lack-of-benefit analysis. The parameter $\theta$ represents the difference in the outcome measure between a research arm and the control group. For continuous outcome measures, $\theta$ could be the difference in the means of the two groups; for binary data the difference in the proportions; for time-to-event data a log hazard ratio. Without loss of generality, assume that a negative value of $\theta _{jk}$ indicates a beneficial effect of treatment *k* in comparison to the control group at stage *j*. In trials with *K* research arms, a set of *K* null hypotheses are tested at each stage *j*,

$$\begin{aligned} H_{jk}^{0}&:&\theta _{jk}\ge \theta _{j}^{0},\ j=1,\ldots ,J \\ H_{jk}^{1}&:&\theta _{jk}<\theta _{j}^{0},\ j=1,\ldots ,J \end{aligned}$$

for some pre-specified null effects $\theta _{j}^{0}$. In practice, $\theta _{j}^{0}$ is usually taken to be 0 on a relevant scale such as the risk (mean) difference for binary (continuous) outcomes or log hazard ratio for survival outcomes [3]. The direction of the hypotheses can be reversed if a trial is seeking an increase in the outcome measure compared to the control arm. For sample size and power calculations, a minimum target treatment effect (often the minimum clinically important difference $\theta _{j}^{1}$) is also required.

At each stage, the significance level $\alpha =(\alpha _1,\ldots ,\alpha _J)$ and power $\omega =(\omega _1,\ldots ,\omega _J)$ are chosen for testing each pairwise comparison of the research treatment *k* against the control group. $L=(l_1,\ldots ,l_{J-1})$ is the lower threshold for (interim) lack-of-benefit on the *Z*-test statistic scale for each pairwise comparion of the research arm *k* against control, determined by $\alpha$ ($l_j=\Phi (\alpha _j)$). The critical value for rejecting the null hypothesis for the selected research arm(s) at the end of the trial is defined as $c=\Phi (\alpha _J)$ - in general, $c=l_J$. A stopping rule for efficacy could also be applied [12, 13]; for simplicity we do not consider it in this article. In the MAMS selection design, an additional selection rule is also pre-specified as $S = (s_1:\ldots :s_{J-1})$, where $s_j$ is the maximum number of research arms to be selected at the end of stage *j*. The selection rule can be written as $K:s_1:s_2:\ldots :s_{J-1}$ reflecting notation by others [8, 14]. Note that $s_{J-1}$ can be greater than one, which means more than one primary hypothesis can be tested at the final stage. However, in practice fewer arms may be selected at the interim stages if not all $s_j$ arms pass the lack-of-benefit threshold.

Let $Z_{jk}= \frac{\hat{\theta }_{jk}}{\sigma _{\hat{\theta }_{jk}}}$ be the *Z*-test statistic comparing research arm *k* against the control arm at stage *j* ($j=1,\ldots ,J$) where $\sigma _{\hat{\theta }_{jk}}$ is the standard error of the treatment effect estimator for comparison k at stage j. $Z_{jk}$ follows a standard normal distribution with the (standardised) mean treatment effect $\Delta _{jk}$, and $Z_{jk} \sim N(0,1)$ under the null hypothesis. The joint distribution of the Z-test statistics therefore follows a multivariate normal distribution:

1 $$\begin{aligned} Z_{11},Z_{12},\ldots ,Z_{JK} \sim MVN(\varvec{\Delta }_{\varvec{JK}}\varvec{,\Sigma }) \end{aligned}$$

where $\varvec{\Delta _{JK}}$ and $\varvec{\Sigma }$ are matrices representing the (standardised) mean treatment effects and the corresponding covariance for the $J\times K$ test statistics, respectively.

At each interim analysis, the test statistics $(Z_{j1},\cdots ,Z_{jk})$ are ranked in order of effect size, denoted by vector $\varvec{\psi }_{\varvec{j}} = (\psi _{j1},\cdots ,\psi _{jK})$, with the rank of research arm *k* at stage *j* given by $\psi _{jk}$ - e.g., the research arm with the largest effect size at stage j will have rank 1, $\psi_{jk} = 1$. An interim decision based on two selection mechanisms is used to determine which research arms should continue to recruit in the subsequent stage:

- If $\psi _{jk} \le s_j \bigcap Z_{jk} \le l_j$, research arm *k* continues to the next stage.
- If $\psi _{jk}> s_j \bigcup Z_{jk} > l_j$, research arm *k* ceases recruitment (‘dropped’).

The operating charactersitics of the design can also be calculated under non-binding interim lack-of-benefit stopping boundaries by replacing $Z_{jk} \le l_j$ ($Z_{jk} > l_j$) with $Z_{jk} \le \infty$ ($Z_{jk} > -\infty$) at interim stages, which effectively means ‘turning off’ the interim stopping boundaries. At the final analysis, the test statistics of the research arms that reached the final stage are compared to the final stage critical value, corresponding to the significance level $\alpha _{J}$, for assessing efficacy:

- If $Z_{Jk} > l_J$, the primary null hypothesis for comparison *k* as before cannot be rejected.
- If $Z_{Jk} \le l_J$, the primary null hypothesis for comparison *k* is rejected and conclude efficacy for research arm *k*.

Next, we outline the steps to design a MAMS selection trial.

### Steps to design a MAMS selection trial

The following steps should be taken to design a MAMS selection trial with interim lack-of-benefit (and efficacy) stopping boundaries.

1. Choose the number of experimental (E) arms, *K*, and stages, *J*. The number of stages should be chosen based on both practical, e.g. expected accrual rate, and statistical considerations [3].
2. Choose the definitive *D* outcome, and (optionally) *I* outcome.
3. Choose the null values for $\theta$ - e.g. the absolute risk difference on the intermediate ($\theta _{I}^{0}$) and definitive ($\theta _{D}^{0}$) outcomes.
4. Choose the minimum clinically relevant target treatment effect size, e.g. in trials with binary outcomes the absolute risk difference on the intermediate ($\theta _{I}^{1}$) and definitive ($\theta _{D}^{1}$) outcomes.
5. Choose the control arm event rate (median survival) in trials with binary (survival) outcome.
6. Choose the allocation ratio *A* (E:C), the number of patients allocated to each experimental arm for every patient allocated to the control arm. For a fixed-sample (1-stage) multi-arm trial, the optimal allocation ratio (i.e. the one that minimizes the sample size for a fixed power) is approximately $A=1/\sqrt{K}$. Choodari-Oskooei et al. provide further guidance for the MAMS selection design when only one research arm is selected at stage 1 [7].
7. In $I\ne D$ designs, choose the correlation between the estimated treatment effects for the *I* and *D* outcomes. An estimate of the correlation can be obtained by bootstrapping relevant existing trial data.
8. Choose the accrual rate per stage to calculate the trial timelines.
9. Choose a one-sided significance level for lack-of-benefit and the target power for each stage ($\alpha _{jk}$, $\omega _{jk}$). The chosen values for $\alpha _{jk}$ and $\omega _{jk}$ are used to calculate the required sample sizes for each stage.
10. Choose whether to allow early stopping for overwhelming efficacy on the primary (*D*) outcome. If yes, choose an appropriate efficacy stopping boundary $\alpha _{Ej}$ on the *D*-outcome measure for each stage 1, ..., *J*, where $\alpha _{EJ}=\alpha _{J}$. Possible choices are Haybittle-Peto or O’Brien-Fleming stopping boundaries used in group sequential designs, or one based on $\alpha$-spending functions - see Blenkinsop et al. 2019 [13] for details.
11. Choose whether to allow for additional treatment selection at interim stages. If yes, choose an appropriate treatment selection rule. For a trial with *J* stages, the selection rule is defined by $K:s_1:s_2:\ldots :s_{J-1}$.
12. Given the above design parameters, calculate the number of control and experimental arm (effective) samples sizes required to trigger each analysis and the operating characteristics of the design, i.e. $n_{jk}$ in trials with continuous and binary outcomes and $e_{jk}$ in trials with time-to-event outcomes, as well as the overall type I error rate and power. If the desired (pre-specified) overall type I error rate and power have not been maintained, for instance if the overall power is smaller than the pre-specified value, steps 9-11 should be repeated until success. Or, if the overall type I error rate is larger than the pre-specified value, one can choose a more stringent (lower) design alpha for the final stage, $\alpha _{J}$, and repeat steps 9-11 until the desired overall type I error rate is achieved.

### Operating characteristics of the MAMS selection design

In this article, we use the term ‘operating characteristics’ to refer to both the overall type I error rate and power. The overarching aim of the MAMS selection design is to reduce the maximum and expected sample size. Therefore, we first define the maximum and expected sample sizes.

#### Maximum and expected sample sizes

The maximum sample size (MSS) is the total sample size for the trial under the assumption that there are $K, s_1, s_2,\ldots , s_{J - 1}$ experimental treatments in each stage - that is, assuming binding treatment selection rules and non-binding lack-of-benefit stopping rules. In the standard (or full) MAMS design, the selection rule is $K, K,\ldots , K$ throughout. Therefore, the maximum sample size is calculated assuming that all experimental treatments continue to the final stage. The expected sample sizes (ESS) under the global null ($H_{0}$) and alternative ($H_{1}$) hypotheses are also calculated for all the simulation scenarios - see Appendix D of the online Supplemental Material for further details and formula. We used simulations to calculate the expected sample sizes.

#### Familywise type I error rate (FWER)

In a MAMS selection design, the research arms are implicitly compared against each other at interim selection stages. This process implicitly links the research arms together. This means that we focus here on the control of the FWER as the type I error rate of interest. Since we consider designs with interim lack-of-benefit analysis, the FWER is the overall probability of a false positive trial result in any of the $s_{J-1}$ comparisons that reach the primary efficacy analysis.

For the standard (keep all promising) MAMS design, the Dunnett probability can be used to calculate the FWER under the global null hypothesis assuming all promising arms are selected [15]. This controls the FWER in the strong sense [16]. Analytical derivations have been developed to calculate the FWER in designs when only one arm is selected for the final stage [8, 17]. However, in the MAMS selection design with more flexible selection rules, the analytical derivations are more complex. Details are included in the online Supplemental Material. In this article, we use simulations to calculate the FWER.

#### Overall power

The power of a clinical trial is the probability that under a particular target treatment effect $\theta ^{1}$, a truly effective treatment is identified at the final analysis. We use simulations to calculate the overall power when one research arm has the target effect size and the other arms have a null effect (i.e. the remaining arms were ineffective). In this case the overall power is defined as the probability that the effective research arm is chosen at the interim selection stages and the primary null hypothesis at final stage is rejected for the comparison of that research arm against the control. This approach to defining power in a multi-arm setting with selection has been adopted by others [18]. Furthermore, we calculate the power to identify any effective research arm (any-pair power) under different configurations of treatment effects and effect sizes - reporting in Appendix E of the online Supplemental Material [11]. Any-pair (or disjunctive) power is the probability that at least one null hypothesis is (correctly) rejected for effective research arms at the final stage.

### Simulation study

Simulations were carried out to explore the impact of the number of research arms selected, the timing of treament selection, and threshold for interim lack-of-benefit analyses on the operating characteristics of a MAMS selection trial. Designs with both binding and non-binding lack-of-benefit stopping boundaries are considered.

#### Trial design parameters

Table 2 presents the trial design parameters in simulation studies. In ROSSINI-2, the first and second interim analyses were scheduled to occur once 21% and 45% of the total control arm patients (that is, information time) were recruited to the trial, respectively. The number of replications is 1,000,000 in each experimental condition. We used Stata 18.0 to conduct all simulations. Further details on the simulation algorithm and the data generating mechanism is included in the online Supplemental Material.

**Table 2:** MAMS selection trial design parameters in the simulation study, based on the ROSSINI-2 trial. Note that 28 different selection rules (including 7:7:7, 7:6:4, 7:5:3, 7:4:2, 7:3:2, 7:3:1) are used under these trial design parameters to explor the effect of different treatment selection strategies

Trial design parameters	Parameter value
Number of research arms	7
Number of stages	3
Stagewise significance level ($\alpha _{j}$)	0.4, 0.14, 0.005
Stagewise pairwise power ($\omega _{j}$)	0.94, 0.94, 0.91
Ctrl. arm sample size at each stage^a^	402, 854, 1887
Ctrl. arm inf. time at stage 1 and 2	0.21, 0.45
Probability of outcome in control arm	0.15
Treatment effect under ${H}_{0}$	0
Treatment effect under ${H}_{1}$	-0.05
Allocation ratio (E:C)	0.5

^a^, nstagebin Stata program is used to obtain sample sizes for each stage - see Appendix B in the online Supplemental Material [10]

Different selection rules were also considered. A factorial approach was followed, testing each parameter in isolation whilst fixing all other parameters of the design. This was done systematically, starting with a design which selects all research arms given they pass the stopping boundary for lack-of-benefit (i.e. the ‘standard’ MAMS design), and decreasing the selected subset size incrementally. Using combinatorics, for a J-stage design there are $$\left( {\begin{array}{c}J+K-1\\ K-1\end{array}}\right)$$ ways of making a subset selection across the $J-1$ interim analyses. For example, for the ROSSINI-2 design, there are 28 ways to select from 7 research arms across two interim analyses.

## Results

### Simulation results

#### Maximum and expected sample sizes

Table 3 presents the required maximum sample size for the primary efficacy analysis by different selection rules. The maximum sample size decreases as the selection rule becomes more strict - that is, when a smaller number of research arms are selected at each stage. For example, it decreases by 49% with the most strict selection rule of 7 : 1 : 1. The maximum sample size for the 7 : 7 : 7 selection rule is the same as that of the standard MAMS design. The expected sample sizes can be substantially lower, depending on the underlying treatment effects of the research arms - see Table 1 in Appendix D of the online Supplemental Material. Next, we describe the impact of the reduction of sample size on the overall operating characteristics of the design.

**Table 3:** The FWER, overall power and maximum sample size for a 8-arm 3-stage trial design, i.e. similar to ROSSINI-2 design by different treatment selection rules - see Table 2 for trial design parameters. The final stage significance level for primary efficacy analysis is $\alpha _{3}=0.005$ in all experimental conditions - with interim stage significance levels of 0.4 and 0.14 at stages 1 and 2, respectively. The overall power is calculated when the primary outcome event risk in one research arm is under the alternative hypothesis and the effect size for the other comparisons is under the null hypothesis. The corresponding expected sample sizes under the global null ($ESS|H_0$) and alternative ($ESS|H_1$) hypotheses are reported in Table 1 of the online Supplemental Material

Performance measure	LOB stopping boundaries	Arms selected at stage 1	Arms selected at stage 2						
			1	2	3	4	5	6	7
FWER	binding	1	0.0125						
		2	0.0167	0.0193					
		3	0.0180	0.0215	0.0220				
		4	0.0193	0.0225	0.0235	0.0239			
		5	0.0196	0.0230	0.0242	0.0246	0.0247		
		6	0.0197	0.0235	0.0245	0.0248	0.0250	0.0250	
		7	0.0200	0.0238	0.0246	0.0249	0.0250	0.0250	0.0250
	non-binding	1	0.0126						
		2	0.0170	0.0210					
		3	0.0193	0.0236	0.0254				
		4	0.0209	0.0252	0.0275	0.0283			
		5	0.0216	0.0262	0.0282	0.0289	0.0301		
		6	0.0219	0.0272	0.0288	0.0296	0.0299	0.0299	
		7	0.0221	0.0274	0.0296	0.0299	0.0303	0.0305	0.0305
Power	binding	1	0.706						
		2	0.792	0.809					
		3	0.816	0.834	0.836				
		4	0.824	0.844	0.846	0.847			
		5	0.827	0.846	0.848	0.849	0.849		
		6	0.827	0.847	0.851	0.849	0.850	0.850	
		7	0.827	0.848	0.850	0.850	0.850	0.850	0.850
	non-binding	1	0.723						
		2	0.825	0.834					
		3	0.858	0.879	0.882				
		4	0.868	0.895	0.898	0.900			
		5	0.873	0.901	0.905	0.906	0.906		
		6	0.875	0.903	0.909	0.909	0.908	0.909	
		7	0.875	0.903	0.908	0.910	0.909	0.910	0.910
Maximum sample size^a^ (final efficacy analysis)		1	4521						
		2	4952	5242					
		3	5285	5624	5963				
		4	5568	5940	6312	6684			
		5	5821	6217	6613	7009	7405		
		6	6056	6470	6884	7298	7712	8126	
		7	6279	6707	7135	7563	7991	8419	8847

^a^The calculations include 4% loss-to-follow-up for the primary outcome in all scenarios

#### Familywise type I error rate and power

Table 3 presents the results for the overall familywise type I error rate and power for different selection rules under the binding and non-binding interim stopping boundaries for lack-of-benefit.

Impact of treatment selection rules: The results indicate that very extreme selection rules (e.g., 7 : 1 : 1) markedly reduces the overall familywise type I error rate under both binding (0.0125) and non-binding (0.0126) interim stopping boundaries for lack-of-benefit. However, the price of this reduced type I error rate is a substantial reduction on the overall power of the trial under the binding (0.706) and non-binding (0.723) interim stopping boundaries for lack-of-benefit. Even selecting 2 arms at the first stage reduces the overall power to 0.79 (from 0.85 for the standard MAMS design) under the binding stopping boundaries for lack-of-benefit. In general, in designs with several research arms, selecting one or two research arms at the first stage selection can decrease the overall power substantially because, given the small sample size, the chance of incorrect selection is high.

An extreme selection rule (e.g., 7 : 1 : 1) can substantially reduce the overall familywise type I error rate. To ensure that the overall type I error for the trial is not underspent, the final stage significance level for the primary analysis can be relaxed in the selection designs with extreme selection rules. Note that in this case the familywise type I error of the selection designs with no interim lack-of-benefit boundaries is still strongly controlled under the global null hypothesis [8]. Although it is intuitive that the FWER will also be maximised for designs with both interim selection rules and lack-of-benefit analysis under the global null hypothesis, this has not been formally proved for designs with both interim selection rules and lack-of-benefit analysis. However, weak control of the FWER is guaranteed at the nominal level.

We used simulations to find the appropriate final stage significance level for the selection designs in Table 3. A grid search was used to find the corresponding value for the final stage significance level in these cases. For the design with 7 : 1 : 1 selection rule, the final stage primary efficacy analysis can be tested at 0.0105 significance level instead of 0.005 level for the standard MAMS design. This further reduces the maximum sample size from 4521 to 4131 for the same overall power of 0.706 and 0.723 under the binding and non-binding interim lack-of-benefit stopping rules, respectively. This results in a further reduction of about 8% - see Table 4 and 5 in Appendix G. Our simulations indicate that for the ROSSINI-2 design with 7 : 5 : 3 selection rule, the final stage significance level of 0.0051 controls the overall FWER at 2.5% (one-sided) - which is very similar (to the fourth decimal place) to that of the standard MAMS design with only interim lack-of-benefit stopping boundaries and a final stage significance level of 0.005. Therefore, the same significance level of 0.005, which is used for final stage primary efficacy analysis in the ROSSINI-2 trial, effectively controls the overall FWER at 2.5% for the standard MAMS design and the ROSSINI-2 selection design. Our simulations have shown that for a MAMS selection design with a selection rule of 7:5:3, the overall operating characteristics of the design are strongly controlled at the pre-specified level with this final stage significance level.

Comparison with the standard MAMS design: The MAMS selection design with $K:K:\cdots :K$ selection rule (i.e. with no restriction on maximum sample size) resembles the standard MAMS design with no selection rule. Results presented in Table 3 indicate that the FWER of the standard MAMS design (Table 1), provides an upper bound for any MAMS selection design with similar design parameters [19, 20].

Here, our aim is to find a candidate MAMS selection design which has similar operating characteristics to that of the ‘optimal’ standard MAMS design. The results in Table 3 indicates that selecting less than 5 research arms at the first stage reduces the overall power of the selection design well below 0.85 - which we targeted for the standard MAMS design. The overall results suggests that a design with 7 : 5 : 3 selection rule gives comparable operating characteristics to that of the optimal standard MAMS design. Table 4 compares different MAMS selection designs with those of the optimal standard MAMS design and two-arm trials. Compared with the optimal standard MAMS design, the selection design with 7 : 5 : 3 selection rule, with a maximum sample size of 6613, decreased the maximum sample size by 25%. Furthermore, this selection design gives the three main interventions the chance to be tested for efficacy at the final analysis if they are selected and pass interim lack-of-benefit boundaries.

**Table 4:** Comparison of the maximum sample sizes and operating characteristics of MAMS selection designs with different selection rules, the standard (or full) MAMS and standard two-arm designs. The design (pairwise) significance levels ($\alpha _j$) and power ($\omega _j$) at stage 1 and 2 are $\alpha =(0.40,0.14)$ and $\omega =(0.94,0.94)$ in the standard MAMS and MAMS selection designs. The final stage significance levels are 0.0105, 0.007, 0.0051 and 0.005 in selection designs no 1, 2, 3, and 4, respectively - see Appendix G in the online Supplemental Material. All sample size calculations assume 4% loss-to-follow-up

Design scenario	Ctrl arm inf. time		Maximum sample size (final analysis)	Overall power	FWER
	Stage 1	Stage 2			
*i*) MAMS selection designs					
1) with 7 : 1 : 1 selection rule	0.21	0.45	4131	0.706	0.025
2) with 7 : 3 : 1 selection rule	0.21	0.45	5108	0.816	0.025
3) with 7 : 5 : 3 selection rule	0.21	0.45	6587	0.848	0.025
4) with 7 : 7 : 7 selection rule	0.21	0.45	8847	0.850	0.025
*ii*) Other designs					
5) Optimal standard MAMS	0.21	0.45	8847	0.850^c^	0.025
6) 7 two-arm trials^a^	–	–	11382	0.850^c^	0.162^b^

^a^two-arm trial with equal (1:1) allocation ratio, each with the one-sided significance level of 0.025 and power of 0.85

^b^overall type I error rate across 7 two-arm trials, $1-(1-0.025)^7$

^c^overall pairwise power

### Timing of treatment selection and early stopping boundaries

This section explores the impact of the timing of treatment selection and interim stopping boundaries for lack-of-benefit on the operating characteristics of a MAMS selection design. The timings of the interim analyses were explored for a range of values of the stagewise significance levels $\alpha _j$ to investigate the impact of the timing of treatment selection on the operating characteristics of the design. This was done by considering different sample sizes (in terms of information time) and significance levels at the interim stages. The other design parameters remained the same.

For stage 1, we considered 10%, 20%, 30% and 40% of the control arm information time - which correspond to stage 1 significance levels ($\alpha _{1}$) of 0.625, 0.42, 0.275, and 0.179, respectively. When varying the timing of the stage 1 analysis, we kept the timing of the stage 2 analysis fixed - that is, at 45% of the control arm information time. For stage 2, we considered 45%, 50%, 60% and 65% of the control arm information time - which correspond to stage 2 significance levels ($\alpha _{2}$) of 0.14, 0.112, 0.07, 0.055, respectively. When varying the timing of the stage 2 analysis, we kept the timing of the stage 1 analysis fixed, that is, at 21% of the control arm information time. We calculated the FWER and overall power under binding interim lack-of-benefit stopping boundaries in all experimental conditions. For brevity, we only present the results for 6 different selection rules. The overall power is calculated when one research arm is effective under the target effect size.

**Fig. 2:**
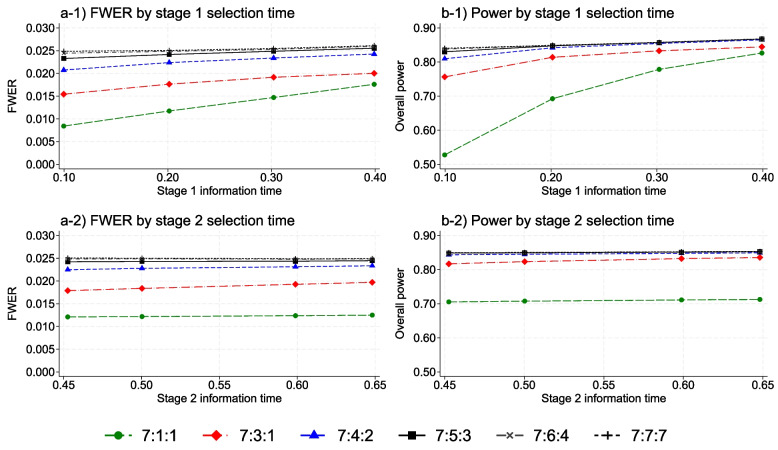
FWER (left) and overall power (right) by the timing of the treatment selection at stage 1 (top) and stage 2 (bottom) and subset selection rule for a three-stage design. The overall power is calculated when one research arm is effective with the target effect size. The X-axis is control arm information time in all graphs

Figure 2 shows the impact of the timing of research arm selection on the FWER and overall power of the MAMS selection design by different selection rules. The top graphs indicate that the timing of the first treatment selection has the most impact on the overall power of a MAMS selection design, since if an efficacious research arm is not selected to continue at the first analysis, the overall power (which is conditional upon selection at stages 1 and 2) cannot be recuperated later. The bottom graphs indicate that the second stage selection time has negligible impact on the operating characteristics of the design.

The FWER and overall power increase by the timing of the first stage treatment selection in all selection rules. Delaying treatment selection may allow more data accrue which support a significantly significant result. However, importantly all choices of significance levels presented here preserve the FWER below the nominal level of 0.025, and result in smaller FWER than that of the standard MAMS design.

The overall power decreases substantially when only one arm is selected at a very early selection stage, i.e., the 7:1:1 selection rule which has an overall power of 0.53 (with a maximum sample size of 3849) when the stage 1 selection takes place at 10% control arm information time. The main reason for the reduced power in this scenario is the high uncertainty associated with the estimated risk by selecting the best performing research arm with a sample size of 94. This reduces the probability of correct selection considerably at the first interim stage which limits the overall power. However, this probability increases considerably when selecting more than 3 research arms at the first interim analysis which results in almost the same overall power as selecting all seven. The maximum sample sizes of other scenarios are included in Table 3 of Appendix F.

## Discussion

In some situations, there is a need to constrain the maximum sample size for a MAMS trial because, for example, there is a limit on the number of patients that can be recruited and/or there is a limited funding envelope for the study. To limit the maximum sample size, an additional pre-specified treatment selection rule can be implemented at interim analyses of a standard MAMS design. This reduces the maximum sample size with minimal impact on the operating characteristics of the trial. Table 4 shows that such a rule can reduce the maximum sample size by about 25% and 42% compared with the optimal standard MAMS design and two-arm trials, respectively. The treatment selection rule acts as an upper bound on the number of research arms that are allowed to continue to the next stage. In practice, depending on how many research arms pass the interim lack-of-benefit analyses, the actual number of research arms that are taken to the next stage might be smaller than the selection rule.

The overall familywise type I error rate of a MAMS selection design is smaller than the corresponding standard MAMS design without a selection rule. It becomes smaller as the selection rule becomes more restrictive. Therefore, investigators may consider relaxing the final stage significance levels for the primary analyses to ensure that the overall type I error for the trial is not underspent. This requires simulations to find the appropriate final stage significance level, which should be done independent of the ongoing trial data, otherwise the overall type I error rate can be inflated over the nominal value [21].

The overall power of a MAMS selection design can be preserved (and remain approximately) at the same level as that of a standard MAMS design if the timing of treatment selection and selection rule are chosen judiciously. The power loss is maximal when only one research arm is selected very early on - i.e., 10% control arm information time in our simulation studies. In this case, to preserve the overall power at above 80%, the timing of the treatment arm selection should be around 40% control arm information time when selecting one effective arm from all possible seven options. In the event more than one arm is to be selected at the first interim analysis, the selection can occur earlier whilst preserving the overall power because the probability that a truly effective research arm is selected is higher at the interim selection stages. This finding accords with previous results [7, 19].

Our simulation results suggest that the choice between the binding and non-binding interim lack-of-benefit stopping boundaries has a larger impact on the overall power. The FWER can increase by 0.005 under the non-binding boundaries, whereas the overall power can decrease by more than 5% under the binding boundaries. Further, there is a pre-specified upper bound on the number of research arms in each stage of a MAMS selection design. Therefore, given the context and the impact on the operating characteristics, binding interim stopping boundaries for lack-of-benefit are more appropriate in this setting. This should be considered when calculating the operating characteristics of a MAMS selection design. Moreover, the impact of varying non-zero treatment effects, smaller than the target effect size, on the overall power is an important design consideration. We conducted extensive simulation studies on this issue. The findings are presented in our previous publication on MAMS selection designs [7].

Finally, the MAMS selection design presented in this article has several advantages over other alternative designs. First, the selection rule is pre-specified and allows for more than one research arm to be selected at the interim stages. Second, the test statistics are based on sufficient statistics, so can be used with covariate adjustment, and also makes the method applicable to different outcome measures. Third, other approaches that allow for more flexible unplanned adaptivity may lose power compared with designs that only allow for pre-planned adaptation if this flexibility is not used in practice [22]. The pre-specification of all adaptations to the design appears to be favoured and recommended by regulators and reviewers [23, 24]. The MAMS selection design satisfies all these considerations. Further, we have implemented the MAMS selection design in the new version of the nstagebin command that is used for sample size calculation. The nstagebin command is available from the Stata’s official archive (ssc) for user written commands. We therefore recommend it as a design to be formally considered in trials in which several research interventions are to be evaluated and where the resources (e.g patients/funding) are limited.

### Supplementary Information

Additional file 1. Online Supplemental Material. Multi-arm multi-stage (MAMS) randomised selection designs: Impact of treatment selection rules on the operating characteristics.

## Data Availability

All data is provided in the main manuscript and its online Supplemental Material. Simulation studies have been used in this article - no real trial data and patient information is used.

## Abbreviations

MAMS: Multi-arm multi-stage
FWER: Familywise type I error rate
ROSSINI-2: The Reduction Of Surgical Site Infection using several Novel Interventions (ROSSINI)-2 trial
