# Correction: Multi‑arm multi‑stage (MAMS) randomised selection designs: impact of treatment selection rules on the operating characteristics

**DOI:** 10.1186/s12874-024-02267-6

**Published:** 2024-07-02

**Authors:** Babak Choodari‑Oskooei, Alexandra Blenkinsop, Kelly Handley, Thomas Pinkney, Mahesh K. B. Parmar

**Affiliations:** 1 MRC Clinical Trials Unit at UCL, Institute of Clinical Trials and Methodology, UCL, 90 High Holborn, London, WC1V 6LJ UK; 2 Department of Mathematics, Imperial College London, London, UK; 3 Birmingham Clinical Trials Unit, University of Birmingham, Birmingham, UK; 4 Institute of Applied Health Research, University of Birmingham, Birmingham, UK

**Correction: BMC Med Res Methodol 24, 124 (2024)**

**https://doi.org/10.1186/s12874-024-02247-w**

Following publication of the original article [1], the authors reported an error found on page 4 and replacement of Fig. 2.

1. On page 4, the following text “\sigma _{\hat{\theta}_{jk}}” should be replaced with ${\sigma }_{{\widehat{\theta }}_{jk}}$.
2. On page 4, the “ψ_jk = 1” should be replaced with ${\psi }_{jk}=1$.
3. In Fig. 2, the title of the two bottom graphs, “a-1” and “b-1” should be replaced with “a-2” and “b-2”, respectively. The revised version of Fig. 2 is shown below.

**Fig. 2:**
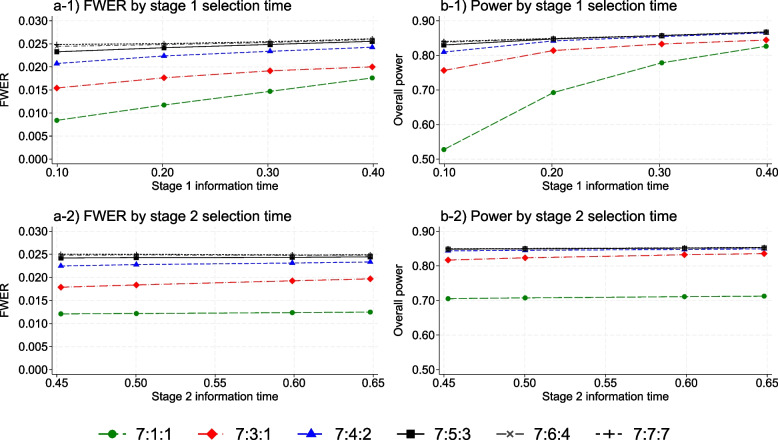
FWER (left) and overall power (right) by the timing of the treatment selection at stage 1 (top) and stage 2 (bottom) and subset selection rule for a three-stage design. The overall power is calculated when one research arm is effective with the target effect size. The X-axis is control arm information time in all graphs

The original article [1] has been updated.
