# Supplementary material for: Multi-arm multi-stage (MAMS) randomised selection designs: impact of treatment selection rules on the operating characteristics
Source: BMC Med Res Methodol. 2024 Jun 3;24:124. doi: 10.1186/s12874-024-02247-w (PMC11145876; doi:10.1186/s12874-024-02247-w)
Supplement: Supplementary file 1 — Additional file 1. Online Supplemental Material. Multi-arm multi-stage (MAMS) randomised selection designs: Impact of treatment selection rules on the operating characteristics. [file 12874_2024_2247_MOESM1_ESM.pdf]

## Online Supplemental Material

# Multi-arm multi-stage (MAMS) randomised selection designs: Impact of treatment selection rules on the operating characteristics

**Babak Choodari-Oskooei, Alexandra Blenkinsop, Kelly Handley, Thomas Pinkney, Mahesh KB Parmar**

**Appendix A) Optimal (admissible) design for ROSSINI-2 MAMS trial**

Admissible MAMS designs are a class of efficient/optimal designs that satisfy the following optimality criteria,

$$L(q) = qE(N|H_1) + (1-q)E(N|H_0) \tag{1}$$

which is a weighted sum of the expected sample size under the global null hypothesis, $E(N|H_0)$, and the hypothesis in which all arms are effective, $E(N|H_1)$.

To find such optimal designs, first a systematic grid-search procedure is conducted over a wide range of the stagewise significance levels and power to find a large set of feasible designs, i.e. designs with a particular (pre-specified) overall type I error rate and power. Then, the procedure selects the most efficient feasible designs, called admissible MAMS designs, using the above optimality criteria proposed by[1]. The corresponding algorithm has been implemented in the `nstagebinopt` Stata command and is available online - further details are included in[2].

Feasible designs which minimise (1) for some $q \in [0, 1]$ are called admissible. Note that the user chooses $q$ based on the prior beliefs about the effectiveness of the treatment under study. Special cases are the null-optimal design with $q = 0$ which minimises the expected sample size under the global null hypothesis, i.e. $E(N|H_0)$, and minimax designs with $q = 1$ which minimises $E(N|H_1)$. However, other admissible designs which minimise a more balanced weighting of the two measures exist.[1] found that these 'balanced' admissible designs are often much more appealing in practice as they usually possess similar desirable properties to the null-optimal or minimax designs but do not have such large maximum or expected sample sizes, respectively. The parameter $q$ could encompass the prior beliefs about the effectiveness of the experimental treatment regimens used in each research arm of the trial or the relative importance of the expected sample sizes under the global null or alternative hypothesis. Designs which minimise the loss function for a wider range of values of $q$ are likely to be more desirable as they are admissible for a wider range of prior beliefs or scenarios. Hence it is important to find the admissible designs for all values of $q$ so that those which cover the broadest range of opinions can be found. The final choice of design will therefore depend on prior beliefs about the effectiveness of the treatment under study, the relative importance of the maximum and expected sample sizes to the investigators or both.

In the ROSSINI-2 trial, the overall familywise type I error rate (FWER) was strongly controlled at 0.025 (one-sided) to account for multiplicity as a result of multiple pairwise comparisons. The overall pairwise power is 0.85. The `nstagebinopt` command used these values to find admissible MAMS designs and determine the corresponding stagewise operating characteristics, $\alpha_j$ and $\omega_j$. Then, the `nstagebin` command calculates the stagewise sample sizes and trial timelines given stagewise (design) operating characteristics that are determined by `nstagebinopt`, as well as all the other design parameters such as the number of arms and stages and control arm event rate.

**Using `nstagebinopt` Stata command to find optimal design**

In multi-arm trials, `nstagebinopt` outputs the stagewise operating characteristics and expected sample sizes under the global null and alternative hypotheses, i.e. $E(N|H_0)$ and $E(N|H_1)$, for each admissible $J$-stage design which minimises

the following loss function $L(q) = qE(N|H_1) + (1-q)E(N|H_0)$ for some $q \in [0,1]$. The program can also save this information in a Stata dataset by specifying the `save()` option. Each admissible design can then be entered into the `nstagebin` program to explore them in more details - see below for the code and its output, i.e. stage durations and sample sizes.

In the design stage of the ROSSINI-2 trial, the control arm SSI rate was assumed to be 0.15, i.e. specified using `ctrlp(0.15)` option in both programs. The trial is powered to detect a target SSI rate of 0.10 in each of the experimental arms, an absolute reduction of $\theta = 5\%$, `theta1(-0.05)`, and relative reduction of 33.3%. Patients are allocated to the control arm with a 2:1 ratio, `aratio(0.5)`, to increase power for each of the pairwise comparisons. In stage 1, which includes the pilot phase, the accrual rate was (on average) assumed to be 118 patients per month. This was expected to increase to 248 patients/month in the subsequent stages, `accrate(118 248 248)`. These recruitment targets were achievable based on the experience with the previous ROSSINI-1 trial. Further, it was assumed that 4% of patients will be lost to follow-up, `ltfu(0.04)`, or the primary outcome evaluation will be missing, e.g. surgery not done. For the interim stage analyses, once the target number of patients is recruited, it was expected that around 4 months will pass until the decision time regarding stopping and/or continuing research arm(s), i.e. `fu(4)`. This was to allow for 30 day follow-up, captured data to be entered, interim analysis to be performed, and Independent Data Monitoring Committee (IDMC) and Trial Steering Committee (TSC) meetings to be held.

The output from `nstagebinopt` is shown below for the ROSSINI-2 MAMS trial with the (one-sided) FWER of 0.025, `alpha(0.025)`, and the pairwise power of 0.85, `power(0.85)`. Note that the FWER is calculated using simulations in both `nstagebin` and `nstagebinopt` commands. For this reason, both programs calculate (and present) the corresponding Monte Carlo error (SE) using the formula $\sqrt{\frac{FWER\times(1-FWER)}{N}}$, where $FWER$ is the calculated overall familywise type I error rate and $N$ is the number of simulations. The range of values of $q$ ($q$-range) for which each design minimises the loss function are also presented. Minimax designs (admissible for $q = 1$) use a high power in the intermediate stages so that the lowest possible power is chosen in the final stage, thus reducing the maximum sample size - see design number 4 in the output. The stagewise powers in the intermediate and final stages then balance out as $q$ decreases (i.e. as $E(N|H_0)$ becomes more of a factor in choosing a design).

The results indicate that the design which is admissible for $q \in [0.10, 0.65]$ has an expected sample size of 4683 patients which is just 25 patients higher than the null-optimal design with 4658 patients. However, this admissible design has a much smaller $E(N|H_1)$ than that of the null optimal design. Overall, this design was selected as the preferred choice. So, the chosen stagewise significance levels and powers were used in the `nstagebin` command for sample size calculations.

```
nstagebinopt, nstage(3) arms(8) alpha(0.025) power(0.85) theta0(0) theta1(-0.05) ///
ctrlp(0.15) ltfu(0.04) fu(4) accrate(118 248 248) aratio(0.5) fwer plot
```

```
n-stage (binary) trial design                    version 1.0.2, 09 June 2023
-------------------------------------------------------------------------------
Admissible designs for a 8-arm 3-stage trial with binary outcome based on
Choodari-Oskooei, Bratton, and Parmar (2023) Stata Journal 23(3).
```

| Design number | q-range | Stage | Sig. level | Power | Alloc. ratio | E(N\|H0) | E(N\|H1) | FWER (SE) |
|---|---|---|---|---|---|---|---|---|
| 1 | [0.00,0.09] | 1 | 0.31 | 0.93 | 0.50 | 4658 | 8667 | 0.0249 |
| | | 2 | 0.16 | 0.93 | | | | (0.0003) |
| | | 3 | 0.005 | 0.92 | | | | |
| 2 | [0.10,0.65] | 1 | 0.40 | 0.94 | 0.50 | 4683 | 8437 | 0.0254 |
| | | 2 | 0.14 | 0.94 | | | | (0.0003) |
| | | 3 | 0.005 | 0.91 | | | | |
| 3 | [0.66,0.77] | 1 | 0.15 | 0.93 | 0.50 | 4989 | 8277 | 0.0258 |
| | | 2 | 0.08 | 0.93 | | | | (0.0003) |
| | | 3 | 0.005 | 0.90 | | | | |
| 4 | [0.78,1.00] | 1 | 0.27 | 0.99 | 0.50 | 6506 | 7824 | 0.0254 |
| | | 2 | 0.14 | 0.99 | | | | (0.0003) |
| | | 3 | 0.004 | 0.85 | | | | |

Note: each design minimises the loss function (1-q)E(N|H0)+qE(N|H1) for values of q specified in q_range. H1 is the hypothesis that all of the experimental arms are effective.

**Appendix B)** `nstagebin` **Stata command to calculate the sample size for ROSSINI-2 trial: optimal standard MAMS design**

This section presents the `nstagebin` command to calculate the required sample size for the ROSSINI-2 MAMS design, together with its output. Most of the design parameters have been defined in Section . The chosen design with the corresponding stagewise significance levels and power from the `nstagebinopt` output are used in the `nstagebin` command to calculate the stagewise sample sizes and timelines. The selected significance levels are 0.40, 0.14 and 0.005 - i.e. `alpha(0.40 0.14 0.005)`. Stages 1 and 2 stagewise significance levels, i.e. 0.40 and 0.14, act as the interim stopping boundaries for lack-of-benefit on the p-value scale. The selected design stagewise powers ($\omega_j$) are 94%, 94% and 91%, respectively for each of the three stages in all 7 pairwise comparisons - `power(0.94 0.94 0.91)`. These stagewise design parameters ensure an overall (one-sided) familywise type I error rate (FWER) of 0.025 and a pairwise power of 0.85.

```
nstagebin, nstage(3) arms(8 8 8) alpha(0.40 0.14 0.005) power(0.94 0.94 0.91) theta0(0) theta1(-
> 0.05) ctrlp(0.15) ltfu(0.04) fu(4) accrate(118 248 248) aratio(0.5) tunit(4) seed(123) ess

n-stage trial design - binary outcome       version 1.0.2, 09 June 2023
-----------------------------------------------------------------------
Sample size for a 8-arm 3-stage trial with binary outcome based on
Bratton et al. (2013) BMC Med Res Meth 13:139 and Choodari-Oskooei,
Bratton, and Parmar (2023) Stata Journal 23(3).
-----------------------------------------------------------------------


Control arm event rate = 0.15
Delay in observing outcome = 4 months
Attrition rate for outcome = 0.04

Operating characteristics
-----------------------------------------------------------------------------
             Alpha(1S)      Power   theta|H0    theta|H1    Length*      Time*
-----------------------------------------------------------------------------
Stage 1         0.4000      0.940      0.000     -0.050     19.979     19.979
Stage 2         0.1400      0.940      0.000     -0.050     10.641     30.620
Stage 3         0.0050      0.910      0.000     -0.050     19.532     50.152
Pairwise        0.0040      0.850                                      50.152
FWER(SE)**      0.0253   (0.0003)
-----------------------------------------------------------------------------
 *  Length (duration of each stage) is expressed in month periods
 **  FWER is calculated using simulations with 250000 replications

Cumulative sample sizes per arm per stage
                       ---------Stage 1---------
```

```
                       Overall  Control   Exper.
------------------------------------------------
Number of active arms        8        1        7
Accrual rate*            118.0     26.2     91.8
Active arms
Patients for analysis     1809      402      201
Patients recruited**      2358      524      262
All arms
Patients recruited**      2358
------------------------------------------------
                       ---------Stage 2---------
                       Overall  Control   Exper.
------------------------------------------------
Number of active arms        8        1        7
Accrual rate*            248.0     55.1    192.9
Active arms
Patients for analysis     3843      854      427
Patients recruited**      4995     1110      555
All arms
Patients recruited**      4995
------------------------------------------------
                       ---------Stage 3---------
                       Overall  Control   Exper.
------------------------------------------------
Number of active arms        8        1        7
Accrual rate*            248.0     55.1    192.9
Active arms
Patients for analysis     8495     1887      944
Patients recruited**      8847     1966      983
All arms
Patients recruited**      8847
------------------------------------------------
 *  Accrual rates are specified in number of patients per month
 ** Accounts for loss-to-follow-up rate and includes those recruited during follow-up periods

Expected sample size | 0 effective arms = 4677
Expected sample size | 7 effective arms = 8435
```

**Appendix C) Familywise type I error rate (FWER), overall power and maximum sample size**

The familywise error rate (FWER) was the type I error measure of interest in this article. The Dunnett probability can be used to calculate the FWER assuming all promising arms are selected (i.e. following the existing MAMS methodology).[3] The FWER can be calculated for the MAMS selection design with interim lack-of-benefit stopping boundaries by the following:

$$\alpha(\boldsymbol{\theta}, c) = \bigcup_k P_{\boldsymbol{\theta}=\mathbf{0}}(Z_{Jk} < l_J, Z_{1k} < l_1 \bigcap \psi_{1k} \leq s_1, Z_{2k} < l_2 \bigcap \psi_{2k} \leq s_2, \ldots, Z_{j-1} < l_{j-1} \bigcap \psi_{j-1k} \leq s_{J-1} | \Sigma, \boldsymbol{\theta_k} \geq 0) \quad (2)$$

where for arm $k$ at stage $j$, $Z_{jk}$ is the cumulative test statistic of the treatment comparison with the control, $\psi_{jk}$ is its rank and $s_j$ is the number of arms selected at interim analysis $j$. $c = l_J$ is the critical value for rejecting the null for the selected research arm at the end of the trial. An example of this expansion, for a special case of the design, given by Lu et al.[4]

**Calculation of the FWER when only one research arm is selected at the final stage**

For a selection design with a 3 : 2 : 1 selection rule and no early stopping, Lu et al.[4] described how to calculate the FWER for normally distributed outcomes ($\theta = \mu_k - \mu_0$) using the following equations. Note that in this setting the direction of the treatvment effect is the opposite to that of the binary outcome in our ROSSINI-2 trial example - i.e., targeting an increase in the continuous outcome. If $\psi = (\psi_1, \psi_2, \psi_3)$ are the rankings of the three research arms, where $\psi_3$ is the research arm that is deselected at the first interim analysis, $\psi_2$ is the research arm deselected at the second analysis, and $\psi_1$ is the research arm which is selected for the final analysis. $c$ is the critical value for rejecting the null for the selected research arm ($\tau$) at the end of the trial, and $Z_{jk}$ is the cumulative standardised treatment effect at stage $j$ for arm $k$. Since there are a small number of permutations of how the research arms could be selected, these can be expanded as

$$\begin{aligned}
\alpha(\boldsymbol{\theta}, c) &= P_{\boldsymbol{\theta}=\mathbf{0}}(Z_{3\tau} > c, \theta_\tau \leq 0) \\
&= \Sigma_{k=1}^{3} P(Z_{3k} > c, \psi_k = 1) I(\delta_k \leq 0) \\
&= P(Z_{31} > c, \psi = (1,2,3)) I(\mu_1 \leq 0) + P(Z_{31} > c, \psi = (1,3,2)) I(\mu_1 \leq 0) \\
&+ P(Z_{32} > c, \psi = (2,1,3)) I(\mu_2 \leq 0) + P(Z_{32} > c, \psi = (3,1,2)) I(\mu_2 \leq 0) \\
&+ P(Z_{33} > c, \psi = (2,3,1)) I(\mu_3 \leq 0) + P(Z_{33} > c, \psi = (3,2,1)) I(\mu_3 \leq 0) \quad (3)
\end{aligned}$$

where $\tau$ is the selected research arm that reaches the final stage. The probability for each of the six permutations can be computed in a similar way - see Lu et al.[4] for further details. The FWER is strongly controlled under the global null hypothesis under the assumption of the equal outcome variance and allocation ratio across research arms.

Note that A key assumption is the equal variance across experimental treatments with equal sample size allocation, or equivalently, the same ratio of variance to sample size for each experimental treatment. If this assumption is violated, the FWER is not necessarily controlled by the type I error rate obtained under the global null hypothesis.

**Overall power**

The power of a clinical trial is the probability that under a particular target treatment effect $\theta^1$, a truly effective treatment

is identified at the final analysis. In multi-arm designs, per-pair (pairwise) power ($\omega$)[5] calculates this probability for a given experimental arm against the control. In multi-arm settings, however, there are other definitions of power that might be of interest, depending on the objective of the trial. These are defined in the following subsections.

In the MAMS selection design, the overall power is the probability that the effective research arm is chosen at the interim selection stages and the primary null hypothesis at final stage is rejected for the comparison of that research arm against the control. As it was evident from the simulation results presented in Results section of the manuscript, a penalty will be incurred on the overall power by restricting the number of arms which transition through the stages of the trial. The overall power depends on the underlying treatment effects. Pairwise power corresponds to the definition of the pairwise error rate under the assumption of the alternative hypothesis (equation 4).

$$\begin{aligned} \omega(\boldsymbol{\theta}, c) = P_{\boldsymbol{\theta}=\boldsymbol{\theta}^1}(Z_{Jk} < l_J, Z_{1k} < l_1 \bigcap \psi_{1k} \leq s_1, Z_{2k} < l_2 \bigcap \psi_{2k} \leq s_2, \ldots, Z_{j-1} < l_{j-1} \\ \bigcap \psi_{j-1k} \leq s_{J-1} | \Sigma, \boldsymbol{\theta_k} < 0) \end{aligned} \tag{4}$$

Disjunctive (or any-pair) power corresponds to the definition of the familywise error rate: the probability of rejecting $H_0$ for any effective research arm.

Many multi-arm studies calculate power under the *least favourable configuration*, which is the probability of rejecting $H_0$ for arm $k$, given it has the target treatment effect and the remaining arms have the minimally clinically relevant treatment effect required to continue investigating the treatment(s)[6]. However in this design, selection of a subset of arms is applied, with the intention of identifying all effective research arms rather than the best performing arm only. Therefore, the procedure does not require specification of the "indifference zone" given by the minimally clinically relevant treatment effect ($\delta^1$) and the effect at which a treatment is considered to be ineffective ($\delta^0$), sometimes termed the "interesting" and "uninteresting" treatment effects, respectively. Because of this, an alternative approach was taken for defining and calculating power. For simulations where only one arm is effective, data for arm $k$ were generated under the target treatment effect, and the remaining arms were generated under the null (i.e. the remaining arms were ineffective). The three measures of power calculated are equal in this setting, and are defined as the probability of rejecting the effective research arm at the final analysis, conditional on its selection at all interim analyses. This approach to define pairwise power in a multi-arm setting with selection has been adopted by others.[7] In Appendix F, we present further simulation results to calculate the overall power under different configurations of treatment effects.

**Appendix D) Sample size**

The maximum sample size (MSS) is the total overall sample size for the trial under the assumption that all research comparisons reach the final stage primary analyses. The maximum sample size is calculated for all design scenarios and reported in this article. The fomulae to calculate the maximum sample size for a MAMS selection design is included in the online Supplemental Material.

The expected sample sizes (ESS) under the global null $(H_0)$ and alternative $(H_1)$ hypotheses are also calculated for all the simulation scenarios - see the online Supplemental Material. The expected sample size takes into account of the probability of $k$ out of $K$ arms passing stage $j$ under treatment effect configuration $\boldsymbol{\theta}$, as well as the additional treatment selection rule.

The maximum sample size (MSS) the MAMS selection design is under the non-binding stopping boundaries. With selection rules, it can be calculated using equation 5.

$$MSS = n_{J0} + s_{J-1}An_{J0} + (s_{J-2} - s_{J-1})An_{J-1\,0} + \ldots, +(s_1 - s_2)An_{20} + (K - s_1)An_{10} \tag{5}$$

where $n_{J0}$ is the cumulative patients in the control arm, $K$ is the number of research arms, $A$ is the allocation ratio between research and control arm and $s_j$ is the number of arms selected at stage $j$.

**Expected sample sizes under the global null and alternative hypotheses**

Table 1 presents the expected sample sizes under the global null $(ESS|H_0)$ and alternative $(ESS|H_1)$ hypotheses by different selection rules for the simulation results presented in Table 4 of the main text.

| Performance measure | LOB stopping boundaries | Arms selected at stage 1 | Arms selected at stage 2: 1 | 2 | 3 | 4 | 5 | 6 | 7 |
|---|---|---|---|---|---|---|---|---|---|
| $ESS\|H_0$ | binding | 1 | 3849 | | | | | | |
| | | 2 | 4119 | 4192 | | | | | |
| | | 3 | 4303 | 4387 | 4407 | | | | |
| | | 4 | 4417 | 4506 | 4538 | 4548 | | | |
| | | 5 | 4477 | 4571 | 4605 | 4625 | 4628 | | |
| | | 6 | 4507 | 4600 | 4640 | 4660 | 4670 | 4671 | |
| | | 7 | 4515 | 4607 | 4655 | 4668 | 4675 | 4678 | 4676 |
| $ESS\|H_1$ | binding | 1 | 4186 | | | | | | |
| | | 2 | 4686 | 4943 | | | | | |
| | | 3 | 5108 | 5431 | 5633 | | | | |
| | | 4 | 5462 | 5825 | 6097 | 6318 | | | |
| | | 5 | 5759 | 6148 | 6485 | 6787 | 7041 | | |
| | | 6 | 6002 | 6411 | 6795 | 7162 | 7499 | 7780 | |
| | | 7 | 6175 | 6595 | 7007 | 7412 | 7800 | 8158 | 8436 |
| $MSS$ | | 1 | 4521 | | | | | | |
| | | 2 | 4952 | 5242 | | | | | |
| | | 3 | 5285 | 5624 | 5963 | | | | |
| | | 4 | 5568 | 5940 | 6312 | 6684 | | | |
| | | 5 | 5821 | 6217 | 6613 | 7009 | 7405 | | |
| | | 6 | 6056 | 6470 | 6884 | 7298 | 7712 | 8126 | |
| | | 7 | 6279 | 6707 | 7135 | 7563 | 7991 | 8419 | 8847 |

**Table 1.** The expected sample sizes for a 8-arm 3-stage trial design, i.e., ROSSINI-2 design[a], by different selection rules under the global null ($ESS|H_0$) and alternative ($ESS|H_1$) hypotheses. The mazimum sample size (MSS) is calculated under the nonbinding interim stopping boundaries for lack-of-benefit. The pairwise design significance level and power are $\alpha = (0.4, 0.14, 0.005)$ and $\omega = (0.95, 0.95, 0.91)$. The calculations include 4% loss-to-follow-up for the primary outcome in all scenarios.

[a] Trial design parameters are presented in Table 2 of the main manuscript. LOB, lack-of-benefit.

**Appendix E) Any-pair power: further simulation results**

This section presents any-pair power for different selection designs by the timing of stage 1 treatment selection. The results are presented in Figure 1 for different configurations of treatment effects and effect sizes: $\boldsymbol{\theta_1}$) three research arms are effective and the remaining arms are under the null, $\boldsymbol{\theta_1}$=(-0.05,-0.05,-0.05,0,0,0,0); $\boldsymbol{\theta_2}$) three research arms are effective and the remaining arms have some partial treatment effect, $\boldsymbol{\theta_2}$=(-0.05,-0.05,-0.05,-0.03,-0.03,-0.03,-0.03); and $\boldsymbol{\theta_3}$) all seven research arms have the target effect, $\boldsymbol{\theta_3}$=(-0.05,-0.05,-0.05,-0.05,-0.05,-0.05,-0.05).

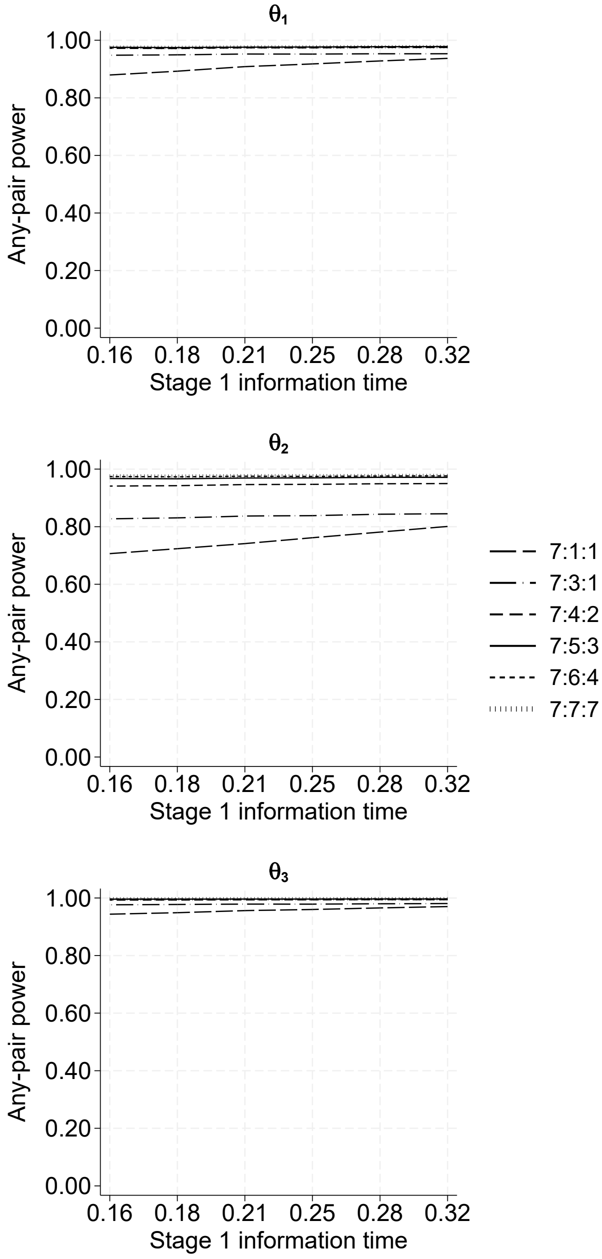


**Figure 1.** Any-pair power under different configuration of treatment effects by each subset selection rule and stage 1 control arm information where $\boldsymbol{\theta_1}$) three research arms are effective and the remaining arms are under the null, $\boldsymbol{\theta_2}$) three research arms are effective and the remaining arms have some partial treatment effect and $\boldsymbol{\theta_3}$) all seven research arms have the target effect.[a]
[a] $\boldsymbol{\theta_1}$=(-0.05,-0.05,-0.05,0,0,0,0), $\boldsymbol{\theta_2}$=(-0.05,-0.05,-0.05,-0.03,-0.03,-0.03,-0.03), $\boldsymbol{\theta_3}$=(-0.05,-0.05,-0.05,-0.05,-0.05,-0.05,-0.05)

**Appendix F) Maximum sample size of the MAMS selection designs by the timing of stage 1 information time**

This section presents the maximum sample sizes of the selection designs by the timing of treatment selection. This was done by considering different information times for treatment selection at interim stages. The other design parameter remained constant. For stage 1, we considered 10%, 20%, 30% and 40% of the control arm information time - which correspond to stage 1 significance levels ($\alpha_1$) of 0.625, 0.42, 0.275, and 0.179, respectively. We calculated the FWER and overall power under binding interim lack-of-benefit stopping boundaries in all experimental conditions and presented them in Figure 2 of the main manuscript. The following table presents the corresponding maximum sample sizes.

| Design $\alpha_j$ | Ctrl. arm sample size ($n_0$) | Inf. time ($t_j$) |
|---|---|---|
| 0.625 | 188 | 0.10 |
| 0.420 | 380 | 0.20 |
| 0.275 | 570 | 0.30 |
| 0.179 | 753 | 0.40 |
| 0.142 | 532 | 0.45 |
| 0.112 | 944 | 0.50 |
| 0.070 | 706 | 0.60 |
| 0.055 | 1223 | 0.65 |
| 0.005 | 1887 | 1.00 |

**Table 2.** The stagewise significance levels, $\alpha_j$, required sample sizes, and the corresponding information times in the control arm for analysis, based on the other design parameters in the ROSSINI-2 trial - see Table 2. The control arm sample size for primary analysis at the final stage is 1887.

| Information time | | Arms selected | Arms selected at stage 2 | | | | | | |
|---|---|---|---|---|---|---|---|---|---|
| Stage 1 | Stage 2 | at stage 1 | 2 | 3 | 4 | 5 | 6 | 7 | 8 |
| | | 1 | 3849 | | | | | | |
| | | 2 | 4392 | 4682 | | | | | |
| | | 3 | 4837 | 5176 | 5515 | | | | |
| 0.10 | 0.45 | 4 | 5232 | 5604 | 5976 | 6348 | | | |
| | | 5 | 5597 | 5993 | 6389 | 6785 | 7181 | | |
| | | 6 | 5944 | 6358 | 6772 | 7186 | 7600 | 8014 | |
| | | 7 | 6279 | 6707 | 7135 | 7563 | 7991 | 8419 | 8847 |
| | | 1 | 4449 | | | | | | |
| | | 2 | 4892 | 5182 | | | | | |
| | | 3 | 5237 | 5576 | 5915 | | | | |
| 0.20 | 0.45 | 4 | 5532 | 5904 | 6276 | 6648 | | | |
| | | 5 | 5797 | 6193 | 6589 | 6985 | 7381 | | |
| | | 6 | 6044 | 6458 | 6872 | 7286 | 7700 | 8114 | |
| | | 7 | 6279 | 6707 | 7135 | 7563 | 7991 | 8419 | 8847 |
| | | 1 | 5043 | | | | | | |
| | | 2 | 5387 | 5677 | | | | | |
| | | 3 | 5633 | 5972 | 6311 | | | | |
| 0.30 | 0.45 | 4 | 5829 | 6201 | 6573 | 6945 | | | |
| | | 5 | 5995 | 6391 | 6787 | 7183 | 7579 | | |
| | | 6 | 6143 | 6557 | 6971 | 7385 | 7799 | 8213 | |
| | | 7 | 6279 | 6707 | 7135 | 7563 | 7991 | 8419 | 8847 |
| | | 1 | 5613 | | | | | | |
| | | 2 | 5862 | 6152 | | | | | |
| | | 3 | 6013 | 6352 | 6691 | | | | |
| 0.40 | 0.45 | 4 | 6114 | 6486 | 6858 | 7230 | | | |
| | | 5 | 6185 | 6581 | 6977 | 7373 | 7769 | | |
| | | 6 | 6238 | 6652 | 7066 | 7480 | 7894 | 8308 | |
| | | 7 | 6279 | 6707 | 7135 | 7563 | 7991 | 8419 | 8847 |

**Table 3.** The maximum sample sizes of different MAMS selection designs by the timing of stage 1 control arm information time. Figure 2 of the main manuscript presents the corresponding FWER (a-1) and power (b-1) of the selection designs.

**Appendix G) Final stage significance levels for primary efficacy analysis to ensure full spending of FWER at 2.5%**

This section includes the results of further simulation studies to find the significance level for the final stage primary efficacy analysis which the controls the overall familywise type I error rate (FWER) at 0.025 (one-sided). Here, the aim is to ensure that the FWER is not underspent.

As the simulation results indicated, the addition of the pre-specified treatment selection rule reduces the FWER of the standard MAMS design. The reduction depends on the selection rule, and how restrictive it is. The more restrict the selection rule is, the larger the FWER reduction is. To ensure that the overall type I error rate is not underspent in some scenarios, particularly in MAMS selection designs with strict selection rules, the final stage significance level can be relaxed such that the FWER is controlled at 2.5% (one-sided). We used simulations to find the final stage significance level that controls the FWER at 2.5% in the 28 different selection designs that are presented in Table 4 of the main text - see Table 4 below. We also calculated the overall power and maximum and expected sample sizes for each scenario under both binding and nonbinding stopping boundaries for lack-of-benefit - see Table 6 and Table 5.

| Design feature | LOB stopping boundaries | Arms selected at stage 1 | Arms selected at stage 2: 1 | 2 | 3 | 4 | 5 | 6 | 7 |
|---|---|---|---|---|---|---|---|---|---|
| Final stage sig. level (FWER: 0.025) | binding | 1 | 0.0105 | | | | | | |
| | | 2 | 0.0077 | 0.0067 | | | | | |
| | | 3 | 0.0070 | 0.0059 | 0.0056 | | | | |
| | | 4 | 0.0066 | 0.0055 | 0.0053 | 0.0052 | | | |
| | | 5 | 0.0065 | 0.0054 | 0.0051 | 0.0051 | 0.0050 | | |
| | | 6 | 0.0065 | 0.0054 | 0.0051 | 0.0051 | 0.0050 | 0.0050 | |
| | | 7 | 0.0065 | 0.0054 | 0.0051 | 0.0051 | 0.0050 | 0.0050 | 0.0050 |
| | non-binding | 1 | 0.0100 | | | | | | |
| | | 2 | 0.0073 | 0.0061 | | | | | |
| | | 3 | 0.0065 | 0.0053 | 0.0050 | | | | |
| | | 4 | 0.0061 | 0.0050 | 0.0046 | 0.0045 | | | |
| | | 5 | 0.0059 | 0.0048 | 0.0044 | 0.0043 | 0.0042 | | |
| | | 6 | 0.0058 | 0.0047 | 0.0043 | 0.0042 | 0.0041 | 0.0041 | |
| | | 7 | 0.0058 | 0.0047 | 0.0043 | 0.0041 | 0.0041 | 0.0041 | 0.0040 |
| Power | binding | 1 | 0.706 | | | | | | |
| | | 2 | 0.792 | 0.809 | | | | | |
| | | 3 | 0.816 | 0.834 | 0.836 | | | | |
| | | 4 | 0.824 | 0.844 | 0.846 | 0.847 | | | |
| | | 5 | 0.827 | 0.846 | 0.848 | 0.849 | 0.849 | | |
| | | 6 | 0.827 | 0.847 | 0.851 | 0.849 | 0.850 | 0.850 | |
| | | 7 | 0.827 | 0.848 | 0.850 | 0.850 | 0.850 | 0.850 | 0.850 |
| | non-binding | 1 | 0.723 | | | | | | |
| | | 2 | 0.825 | 0.834 | | | | | |
| | | 3 | 0.858 | 0.879 | 0.882 | | | | |
| | | 4 | 0.868 | 0.895 | 0.898 | 0.900 | | | |
| | | 5 | 0.873 | 0.901 | 0.905 | 0.906 | 0.906 | | |
| | | 6 | 0.875 | 0.903 | 0.909 | 0.909 | 0.908 | 0.909 | |
| | | 7 | 0.875 | 0.903 | 0.908 | 0.910 | 0.909 | 0.910 | 0.910 |

**Table 4. Top**: The final stage significance level for the final primary efficacy analysis that controls the FWER at 0.025 (one-sided) in a 8-arm 3-stage trial design, i.e., ROSSINI-2 design[a], by different selection rules and binding/non-binding interim lack-of-benefit stopping boundaries. Other design parameters remain the same and presented in Table 2 of the main text. LOB, lack-of-benefit.
**Bottom**: The overall power of the selection designs given the final stage significance level at the top.

| Performance measure | LOB stopping boundaries | Arms selected at stage 1 | Arms selected at stage 2 1 | 2 | 3 | 4 | 5 | 6 | 7 |
|---|---|---|---|---|---|---|---|---|---|
| | | 1 | 4131 | | | | | | |
| | | 2 | 4726 | 5038 | | | | | |
| | | 3 | 5108 | 5508 | 5863 | | | | |
| $MSS$ | binding | 4 | 5430 | 5872 | 6261 | 6642 | | | |
| | | 5 | 5683 | 6164 | 6578 | 6985 | 7405 | | |
| | | 6 | 5918 | 6417 | 6864 | 7274 | 7712 | 8126 | |
| | | 7 | 6141 | 6654 | 7115 | 7539 | 7991 | 8419 | 8847 |
| | | 1 | 4157 | | | | | | |
| | | 2 | 4753 | 5102 | | | | | |
| | | 3 | 5147 | 5583 | 5963 | | | | |
| $MSS$ | nonbinding | 4 | 5463 | 5940 | 6386 | 6792 | | | |
| | | 5 | 5734 | 6244 | 6722 | 7165 | 7615 | | |
| | | 6 | 5978 | 6510 | 7014 | 7478 | 7950 | 8398 | |
| | | 7 | 6201 | 6747 | 7265 | 7767 | 8229 | 8691 | 9197 |

**Table 5.** The maximum sample sizes ($MSS$) for selection designs with interim binding/nonbinding lack-of-benefit stopping boundaries in Table 4. The calculations include 4% loss-to-follow-up for the primary outcome in all scenarios.

| Performance measure | LOB stopping boundaries | Arms selected at stage 1 | Arms selected at stage 2 1 | 2 | 3 | 4 | 5 | 6 | 7 |
|---|---|---|---|---|---|---|---|---|---|
| | | 1 | 3748 | | | | | | |
| | | 2 | 4039 | 4121 | | | | | |
| | | 3 | 4231 | 4342 | 4379 | | | | |
| $ESS\|H_0$ | binding | 4 | 4356 | 4477 | 4519 | 4538 | | | |
| | | 5 | 4418 | 4547 | 4595 | 4616 | 4629 | | |
| | | 6 | 4446 | 4576 | 4632 | 4650 | 4665 | 4667 | |
| | | 7 | 4455 | 4585 | 4642 | 4661 | 4676 | 4679 | 4679 |
| | | 1 | 4004 | | | | | | |
| | | 2 | 4527 | 4790 | | | | | |
| | | 3 | 4961 | 5331 | 5553 | | | | |
| $ESS\|H_1$ | binding | 4 | 5336 | 5762 | 6052 | 6283 | | | |
| | | 5 | 5626 | 6096 | 6452 | 6765 | 7041 | | |
| | | 6 | 5867 | 6357 | 6776 | 7139 | 7498 | 7780 | |
| | | 7 | 6038 | 6541 | 6987 | 7387 | 7800 | 8158 | 8437 |

**Table 6.** The expected sample sizes under the global null ($ESS|H_0$) and alternative ($ESS|H_1$) hypotheses by binding interim lack-of-benefit stopping boundaries for the selection designs in Table 4. The calculations include 4% loss-to-follow-up for the primary outcome in all scenarios.

**Appendix H) Data generating mechanism in the simulation studies**

For binary outcomes, $X$ is the observed response for each patient in the control arm (0) and research arm $k$:

$$X \overset{\text{iid}}{\sim} Ber(\pi_0)$$
$$X \overset{\text{iid}}{\sim} Ber(\pi_k)$$

$Y_{j0}$ and $Y_{jk}$ are the observed number of responses in the control arm and research arm $k$ at stage $j$, respectively. $n_{jk}$ denotes the number of patients recruited to arm $k$ between stages $j-1$ and $j$, and $\pi_0$, $\pi_k$ the event rates in the control and research arms.

$$Y_{j0} \sim Bin(n_{j0}, \pi_0)$$
$$Y_{jk} \sim Bin(n_{jk}, \pi_k)$$

Data was generated for the different stages independently under the binomial distribution. At stages 2 to $J$, the data was added cumulatively to the previous stages, inducing the correlation between treatment effect estimates at different stages of the same pairwise comparisons. Correlation between the arms was also induced through the use of the shared control arm in calculating test statistics of each treatment comparison. The data generating mechanism was validated by verifying the empirical correlation against the expected theoretical values.

The event rate of SSI in each research arm $k$, and the control arm, at stage $j$ were calculated as:

$$\hat{\pi}_{jk} = \frac{\hat{Y}_{jk}}{N_{jk}}$$
$$\hat{\pi}_{j0} = \frac{\hat{Y}_{j0}}{N_{j0}}$$

where $N_{jk} = \sum_{l=1}^{j} n_{lk}$ is the cumulative number of patients recruited to the trial by stage $j$ in arm $k$.

The treatment effect being estimated at the end of the trial for the binary case is defined by the risk difference: $\theta_k = \pi_k - \pi_0$. Since the trial is looking to detect interventions which reduce the proportion of post-surgery site infections, a negative treatment effect indicates benefit of a research arm over the control arm.

In the event of ties of test statistics, which will generally only occur in trials with small sample sizes, and at the first stage interim analysis, others have suggested a preference rule should be established when starting the trial, for example based on safety and cost profiles of the arms, to handle ties due to difficulties in obtaining unbiased estimators.[8,9]

## References


1. Jung SH, Kim KM. On the estimation of the binomial probability in multistage clinical trials. Statistics in Medicine. 2004 mar;23(6):881–896. Available from: http://doi.wiley.com/10.1002/sim.1653.
2. Choodari-Oskooei B, Bratton D, Parmar MKB. Facilities for optimizing and designing multiarm multistage (MAMS) randomized controlled trials with binary outcomes. The Stata Journal. 2023;23(3):744–798. Available from: https://doi.org/10.1177/1536867X231196295.
3. Dunnett CW. A Multiple Comparison Procedure for Comparing Several Treatments with a Control. Journal of the American Statistical Association. 1955;50(272):1096–1121.
4. Lu X, He Y, Wu SS. Interval estimation in multi-stage drop-the-losers designs. Statistical Methods in Medical Research. 2018;27(1):221–233. Available from: https://journals.sagepub.com/doi/pdf/10.1177/0962280215626748.
5. Royston P, Barthel FMS, Parmar MKB, Choodari-Oskooei B, Isham V. Designs for clinical trials with time-to-event outcomes based on stopping guidelines for lack of benefit. Trials. 2011;12(1):81. Available from: http://www.trialsjournal.com/content/12/1/81.
6. Dunnett CW. Selection of the Best Treatment in Comparison to a Control with an Application to a Medical Trial. In: Santer T, Tamhane A, editors. Design of Experiments: Ranking and Selection. New York: Marcel Dekker; 1984. p. 47–66.
7. Kunz CU, Friede T, Parsons N, Todd S, Stallard N. A comparison of methods for treatment selection in seamless phase II/III clinical trials incorporating information on short-term endpoints. Journal of Biopharmaceutical Statistics. 2015;25(1):170–189. Available from: https://www.tandfonline.com/action/journalInformation?journalCode=lbps20.
8. Tappin L. Unbiased estimation of the parameter of a selected binomial population. Communications in Statistics - Theory and Methods. 1992 jan;21(4):1067–1083. Available from: http://www.tandfonline.com/doi/abs/10.1080/03610929208830831.
9. Luo X, Wu SS, Xiong J. Parameter estimation following an adaptive treatment selection trial design. Biometrical Journal. 2010 dec;52(6):823–835. Available from: http://doi.wiley.com/10.1002/bimj.200900134.
